# Integrated Dynamic Autonomic and Cardiovascular Regulation during Postural Transitions in Older Adults Living with Frailty: A Systematic Review Protocol

**DOI:** 10.3390/ijerph20010566

**Published:** 2022-12-29

**Authors:** Dihogo Gama de Matos, Jefferson Lima de Santana, Asher A. Mendelson, Todd A. Duhamel, Rodrigo Villar

**Affiliations:** 1 Cardiorespiratory & Physiology of Exercise Research Laboratory, Faculty of Kinesiology and Recreation Management, University of Manitoba, Winnipeg, MB R3T 2N2, Canada; 2 Section of Critical Care, Department of Medicine, Rady Faculty of Health Sciences, University of Manitoba, Winnipeg, MB R3E 0W2, Canada; 3 Faculty of Kinesiology and Recreation Management, University of Manitoba, Winnipeg, MB R3T 2N2, Canada; 4 Institute of Cardiovascular Sciences, St. Boniface General Hospital Albrechtsen Research Centre, Winnipeg, MB R2H 2A6, Canada

**Keywords:** older adults, frailty, dysautonomia, cardiovascular dysfunction, postural transition

## Abstract

Older adults often experience episodes of a sudden drop in blood pressure when standing, known as orthostatic hypotension (OH). OH is associated with an increased risk of life-threatening health problems, falls, and death. Although OH has been studied in older adults, the integrated dynamic autonomic and cardiovascular regulation during postural transitions in older adults with frailty remains scarce and poorly understood. The primary aim of this systematic review is to determine the association between how active (e.g., lie-to-stand) and passive (head-up tilt) postural transitions affect the dynamic integrated autonomic and cardiovascular regulatory responses, comparing older adults with different levels of frailty (non-frail, pre-frail, or frail). A second aim is to perform a meta-analysis to compare autonomic and cardiovascular responses during active postural transitions in non-frail, pre-frail, and frail older adults. The systematic review will be outlined according to the Preferred Reporting Items for Systematic Review and Meta-Analysis Protocols. The meta-analysis will generate estimates of the comparative autonomic and cardiovascular responses after active postural transitions in adults who are non-frail, pre-frail, and frail. This systematic review will provide critical information on how integrated dynamic autonomic and cardiovascular regulation occurs during postural transitions in older adults with different frailty statuses.

## 1. Introduction

Frailty is characterized by a decline in biological functional reserve to tolerate stressors and increases the risk of several physiological deficits [1]. Although significantly associated with aging [1], it is more complex, and it is not directly determined by chronological age. In fact, people of all ages can be frail [2]. Frailty is a vicious cycle influenced by several physiological, cultural, and social factors [3,4,5,6]. In 2020, over 1.6 million Canadians aged 65+ were diagnosed with frailty [7,8]. These numbers are expected to rise to 2.5 million in ten years. Currently, $220 billion is expended annually on health care in Canada, where 46% is spent on individuals over 65 years of age [8]. Although more than 51% of community-dwelling older adults are considered frail (10%) and pre-frail (41%) [1], they are under-recognized, under-served, under-appreciated, and poorly understood [8]. Frailty is also associated with difficulties in performing daily activities (e.g., postural transitions), adverse health consequences (e.g., chronic diseases) [1], and life-threatening hazards (e.g., falls) [9]. It has also been linked to autonomic and cardiovascular dysregulation [8,9], as it compromises the rapid adjustments of these integrative regulatory mechanisms.

Dynamic control and regulation of the autonomic and cardiovascular systems is required for human survival. The integration of these physiological systems regulates homeostasis and adequate functioning in the face of external stressors [10,11,12]. The stress induced by postural transitions (e.g., standing from a bed or chair) evokes a range of quick adaptive physiological responses such as an increase in heart rate and total peripheral resistance to maintain cardiac output, cerebral pressure, and cerebral blood flow [11]. If the physiological integrations of the autonomic and cardiovascular systems are not well adjusted to reach the task demands, people will struggle to perform even activities of daily living (ADLs) [13]. Impairment/dysfunction of these physiological systems exposes people to higher risks of frailty, chronic diseases, and falls [14,15]. Therefore, the autonomic and cardiovascular systems orchestrate tight and complex control, and regulation plays a critical role in matching physiological demands and supply for homeostasis maintenance and appropriate physiological system function [11]. However, homeostatic dysregulation compromises these rapid adjustments in the autonomic and cardiovascular regulatory mechanisms. This homeostatic dysregulation is most prevalent in more vulnerable populations, such as older adults, and might be further compromised in the presence of frailty [16,17].

Orthostatic hypotension is a drop in systolic and diastolic blood pressure that occurs when assuming an upright position [18]. Nearly one in four older adults (25%) are diagnosed with OH, but one-third are asymptomatic [19]. The symptoms may include dizziness, blackouts, blurred vision, dyspnea, angina pectoris, paracervical and lumbar pain, weakness, and nausea [20,21]. OH has been associated with postural instability, falls, pre-syncope, syncope, functional impairment, cardiovascular events, and increased mortality in older adults [18]. Orthostatic hypotension occurs when autonomic responses mediated by baroreceptors are insufficient to maintain blood pressure when standing or when blood volume is inadequate to sustain ventricular filling [22]. A reduction in baroreceptor sensitivity, autonomic dysfunction, sarcopenia, hypovolemic disorders, and prolonged bed rest are all thought to be potential causes of OH [22].

The possible explanations for the higher prevalence of OH in older adults are associated with baroreflex sensitivity deterioration, higher prevalence of comorbidities, and medications [23,24]. Older adults are more likely to develop orthostatic intolerance (inability to respond to the challenge imposed by the upright posture) [25]. Research suggests that orthostatic intolerance symptoms (e.g., dizziness, light-headedness, and nausea) are associated with frailty [26]. Therefore, individuals with frailty may have a compromised ability to adapt to situations that require faster autonomic and cardiovascular adjustments (e.g., lie-to-stand). Most of the studies published so far have focused on whether there is a relationship between frailty and OH. For example, Kocyigit et al. [27] showed that frailty is associated with OH, particularly in the first minute after head-up tilt test. Liguori et al. [28] indicated that OH is a common condition in frail older adults ranging from 9% to 66% incidence depending on the degree of frailty. Romero-Ortuno et al. [29] reported that pre-frail and frail older adults had the capacity to recover systolic blood pressure within 30 s after standing (pre-frail = 95%; frail = 92% recovery). Despite suggestions that there is a relationship between frailty and OH, little is known about the autonomic and cardiovascular regulation during postural transitions in this population.

The assessment of variables (systolic and diastolic blood pressure) to provide a proper understanding of OH is performed through an active standing test (AST, e.g., lie-to-stand) or a passive head-up tilt test (HUT). Although these two tests are commonly used, there is no consensus on which test is the most appropriate and effective for diagnosing OH. For example, the HUT is a widely used test in clinical settings. However, a HUT requires costly specialized equipment (a tilting table), is time-consuming, requires adequate training in the use of equipment, sometimes gives a false positive result, do not mimic real life activity, and cannot detect initial orthostatic hypotension due to the gradual increase in the angle of tilting. The active standing does not require specialized equipment, it is easy to perform, requires less training, represents a very common activity of daily living, detects initial OH, and is reliable [30,31]. However, this test is not suitable for people who have immobility (e.g., spinal cord injury). Even though such tests can be used to diagnose OH, little is known about the comparison between these two tests involving the frailty population.

Oyake et al. [31] evaluated 19 healthy young adults and compared hemodynamic responses between the AST and the HUT. The authors observed that systolic blood pressure (SBP) and heart rate (HR) were higher and stroke volume (SV) lower during AST compared to HUT. According to the authors, these responses may be associated with an increase in total peripheral resistance (TPR). Aydin et al. [32] analyzed 290 geriatric patients in both tests, showing that the AST had a high prevalence of OH (37%) compared to the HUT (19%). Therefore, it is necessary to compare both tests to better understand the hemodynamic differences and their implications to OH diagnosis, particularly in people with different frailty levels. A comprehensive systematic literature review is needed in order to better understand the integrated dynamic autonomic and cardiovascular responses during postural transitions (orthostatic stress) in older adults with different frailty statuses, particularly people living with frailty. In this systematic review, we will determine the association between the integrated dynamic autonomic and cardiovascular regulatory responses in older adults during postural transitions (active and passive) and frailty levels (non-frail, pre-frail, or frail), and we will perform a meta-analysis to compare autonomic and cardiovascular responses during the active postural transition in non-frail, pre-frail, and frail older adults.

## 2. Materials and Methods

This systematic review protocol was registered in the PROSPERO International database of prospectively registered systematic reviews (protocol #: CRD42022268613). It is based on the Population Intervention Comparison Outcome (PICO) framework [33]. Based on this framework, the population of interest for this review is non-frail, pre-frail, and frail older adults (65+ years) from the community, clinics, hospitals, and nursing homes. The exposure of interest is frailty status, and the outcome of interest is the autonomic and cardiovascular responses during postural transitions. We are also following the Preferred Reporting Items for Systematic Review and Meta-Analysis protocol (PRISMA-P) reporting guideline for systematic review protocols [34].

### 2.1. Search Strategy

The PICO approach was used to define the primary research questions and help to formulate the search strategy: (P) Population: non-frail, pre-frail, and frail older adults aged 65 years or older; (I) Intervention: postural transitions (active postural transition and head up-tilt test); (C) Comparison: autonomic and cardiovascular responses during postural transitions in non-frail, pre-frail, and frail older adults; (O) Outcomes: understand how autonomic and cardiovascular systems respond to orthostatic stress challenges (postural transitions). Therefore, the research question is as follows: “*In non-frail, pre-frail, and frail individuals, how do postural transitions (orthostatic stress) affect the dynamic integrated autonomic and cardiovascular regulatory responses?*”

**Figure 1:**
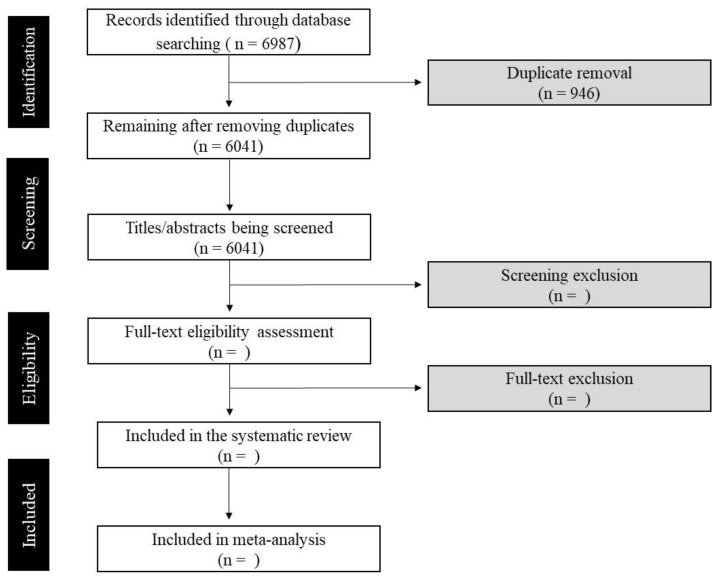
Study identification and screening flow chart.

MEDLINE, PubMed, CINAHL, SCOPUS, and EMBASE databases were systematically searched in October 2022. A customized search strategy was created by DGM for each database, including keywords and MeSH terms based on previously published systematic reviews [30,35,36] with support of the University librarian. The search strategy included the keywords ‘frailty’, ‘orthostatic hypotension’, ‘postural transition’, ‘older adults’, ‘aging’, and ‘blood pressure’ (Appendix A). The searches were based on investigations of frailty, OH, and hemodynamics variables (systolic and diastolic blood pressure, cardiac output, heart rate, and heart rate variability) in cohorts of older adults (≥65 years). We did not apply date restrictions in any of our searches. The search using the selected keywords resulted in 6987 studies. However, 946 studies were duplicated, leaving 6041 for the first screening. Figure 1 shows the flow diagram illustrating our approach.

### 2.2. Study Selection Criteria

Studies are being organized and managed using Covidence systematic review software (Veritas Health Innovation, Melbourne, Australia). Two researchers (DGM and JLS) are reviewing the selected manuscript titles and abstracts. An exclusion and inclusion criteria will be used to remove the manuscripts that do not fit the established criteria, and the reviewers will fully screen the remaining papers. The inclusion criteria were the following: (1) older adults (≥65 years) classified as non-frail, pre-frail, and frail; (2), Fried Phenotype and Frailty Index (minimum of 30 questions) must have been used to define and classify frailty statuses; (3) presence of a control group (non-frail); (4) blood pressure must have been monitored during the whole experiment; (5) blood pressure, heart rate, heart rate variability considered as parameter of interest; (6) experimental, case-control, and cross-sectional studies; and (5) articles published in English. The exclusion criteria included the following: (1) quasi-experimental design, reviews, meta-analysis, conference abstracts, editorials, and letter to the editor; (2) participants with severe cognitive impairments, neurodegenerative disorders (e.g., Alzheimer’s, Parkinson’s, pure autonomic failure); and (3) animal studies.

### 2.3. Data Extraction

Two researchers (DGM and JLS) independently extract data. The full text of these potentially eligible studies, when available and relevant, will be extracted and compiled as follow: the leading author, country, year of publication, study design, population studied, total sample size, age of participants, type of postural transition (active and/or passive), and OH classification will be detailed in a table format (Table 1). If there is any disagreement regarding specific studies’ eligibility, a third researcher (RV) will resolve this issue.

We will utilize the definition of OH as follow: (1) initial OH (systolic blood pressure drop > 40 mmHg or diastolic blood pressure drop > 20 mmHg in the first 15 s when assuming an upright position) [18]; (2) classical OH (systolic blood pressure drop > 20 mmHg or diastolic blood pressure drop > 10 mmHg between 30 to 180 s when assuming an upright position) [18]; and (3) delayed OH (the progressive fall in systolic blood pressure after 3 min when assuming an upright position) [18]. For frailty status, we are using the Frailty Phenotype [3] or Frailty Index [37] (minimum 30 variables including signs, diseases, disabilities, and symptoms) for classification purposes. For the systematic review, we will extract data from studies that published either active (e.g., sit-to-stand, lie-to-stand, and lie-to-stand) and passive (head-up tilt) postural transitions with continuous monitoring of beat-by-beat blood pressure for further analysis (aim 1). For the meta-analysis (aim 2), only the active postural transitions will be used to extract information for further meta-analysis. We selected only active postural transitions for the meta-analysis because active postural transitions are prevalent activities of daily living, which is not the case with passive postural transitions (HUT). During active standing, the skeletal muscle is activated, and the muscle contractions support venous return by compressing capacitance vessels preventing excessive accumulation of blood in the lower body [38], which does not occur during passive tilting. There is a higher prevalence of orthostatic hypotension during active postural transition compared to the passive postural transition (37% and 19%, respectively) [16]. Lastly, during the head-up tilting test (passive postural transition), initial orthostatic hypotension cannot be detected due to the gradual rise of the titling [39]; thus, passive tilting does not replicate the physiology of the acute act of standing [40].

### 2.4. Study Quality and Risk of Bias Assessments

Two independent reviewers (DGM and JLS) will assess the quality and bias of each included article using the nine-point Newcastle–Ottawa Scale (NOS) [41]. A third senior author (RV) will resolve disagreements if they occur. Articles with a NOS score between 0 and 3 points will be classified as low quality, 4–6 as moderate quality, and 7–9 as high quality [41]. We have chosen the NOS scale because it assesses several criteria in three domains that will be used in this systematic review: (1) selection, (2) comparability, and (3) outcome. Additionally, this scale has been used in previous systematic reviews on related topics such as frailty [42] and orthostatic hypotension [35].

### 2.5. Study Selection for Meta-Analysis

Meta-analysis will be performed if at least two studies match the following selection criteria: (1) studies had a similar study design (e.g., experimental studies, presence of control group, active or passive postural transitions); (2) the population is non-frail, pre-frail or frail older adults, 65 years or older; (3) blood pressure was measured continuously (e.g., Finometer Finapress); (4) studies used an active postural transition as an orthostatic stress test; (5) studies have systolic and diastolic blood pressure, heart rate, cardiac output, and heart rate variability analyzed; (5) heart rate variability was collected via an electrocardiogram (ECG) and in the frequency domain: (1) low frequency band 0.04–0.15 Hz, and (2) high-frequency band 0.15–0.40 Hz.

### 2.6. Data Synthesis

The following data will be extracted: (1) continuously systolic and diastolic blood pressure, cardiac output, heart rate, and heart rate variability measurements and (2) results of the active stand test and head-up tilt test (passive test). The study population will be categorized as non-frail, pre-frail, and frail according to the Fried Phenotype classification [3]. The meta-analysis will use a random–effect model, including at least two studies using the Review Manager software (RevMan. Version 5.3. Copenhagen, Denmark: The Nordic Cochrane Centre, The Cochrane Collaboration) [43]. The means and standard deviations will be used to compute the standardized mean difference (SMD) for the retrieved continuous variables. Analysis of heterogeneity effects will be performed using I^2^ statistics, where values of heterogeneity < 25% will be considered low, <50% moderate, and >50% high [44]. Sensitivity analysis will be performed in case of unclear statistical discrepancy observed by removing those studies from the analysis. The publication bias will be assessed through Eggers’s test for meta-analyses using a significant level of 10% with at least 10 studies [45].

## 3. Discussion

This will be the first systematic review seeking to report how the postural transition may affect the dynamic integrative autonomic and cardiovascular regulatory responses in people with different frailty levels. We will be able to rigorously summarize, synthesize, and compare the results and characterize the main variables associated with physiological dysfunction in frailty. This systematic review will build on original studies involving analyzes during active and passive postural transitions (orthostatic stress test) to obtain a more comprehensive view of physiological responses during external stressors. Studies that use the active postural transition will be analyzed in the meta-analysis to obtain a physiological response within daily reality and the ability to detect initial orthostatic hypotension.

Another strength of this study is the comparison of the non-frail, pre-frail, and frail groups. No previous systematic review has compared the autonomic and cardiovascular responses during the postural transitions in those groups. Few studies focus on pre-frail and frail older adults [27], since most of the original studies focus on analyzing the physiological responses to orthostatic stress in groups of older, healthy adults [46,47] and chronic diseases [48,49]. Moreover, most studies that analyze frailty do not focus on the dynamic integrative autonomic and cardiovascular regulatory responses to postural transitions [27]. Therefore, this systematic review is extremely necessary, and the findings obtained will inform how individuals living under pre-frail or frail conditions adapt to orthostatic stress challenges, opening relevant directions for new investigations, insights, and research endeavours.

## 4. Conclusions

With the results of this systematic review, we will be able to provide critical information by synthetizing the current evidence on the association between the integrated dynamic autonomic and cardiovascular regulatory responses in older adults with different levels of frailty during postural transitions. We will also provide comparisons between non-frail, pre-frail, and frail older adults regarding their autonomic and cardiovascular responses during active postural transition.

## Figures and Tables

**Table 1:** Variables to be extracted at the full text.

Bibliographic	Title Authors Year of publication Study design Population studied Country Setting(s) Inclusion and exclusion criteria outlined by the study
Demographics	Age Sample size Description of health status
Postural transition	Active postural transition Passive postural transition (head-up tilt test)
Frailty assessment	Non-frail, pre-frail and frail classification based on Frailty Phenotype and Frailty Index
Orthostatic hypotension	Initial orthostatic hypotension Classical orthostatic hypotension Delayed orthostatic hypotension
Blood pressure measurements	Continuous (e.g., Finometer Finapress)
Hemodynamic variables	Blood pressure (systolic, diastolic, mean arterial pressure) Heart rate Cardiac output
Autonomic nervous system	Heart rate variability

## Data Availability

Not applicable.

## Appendix A. Search Strategy

**Table:**

MEDLINE Search Strategy		
	Search string	Results
1	Frail Elderly/	13,245
2	frailty.mp. or Frail Elderly/or Frailty/	26,263
3	(frail* or pre frail* or prefrail*).mp	32,642
4	1 or 2 or 3	32,642
5	Aging/	240,064
6	(ageing or elderly or old or geriatric or elder*).mp	1,477,953
7	4 or 5 or 6	1,642,541
8	Hypotension, Orthostatic/	5896
9	(hypotension or hypotens* or blood pressure or drop blood pressure).mp	519,299
10	(postural transition or head-up test or postural challenge or standing or stand).mp	123,467
11	7 and 8 and 9	**1042**
**EMBASE Search Strategy**		
	Search string	Results
1	(frailty or frail elderly).mp	39,661
2	frailty/or frailty.mp	30,899
3	(frail* or pre frail or prefrail*).mp.	48,182
4	1 or 2 or 3	48,182
5	(ageing or aging or aged or elderly or old).mp.	6,416,913
6	4 or 5	6,426,419
7	orthostatic hypotension.mp. or orthostatic hypotension/	23,365
8	(hypotension or hypotense* or drop blood pressure).mp.	190,445
9	(postural transition or head-up test or postural challenge or standing or stand).mp.	169,445
10	6 and 7 and 9	**1525**
**CINAHL Search Strategy**		
	Search string	Results
S6	S3 AND S4 AND S5	**716**
S5	(postural transition or postural challenge) OR (sit to stand or sts or sit-to-stand) OR (lie to stand or standing) OR ( position* or transition*)	200,622
S4	(orthostatic hypotension or postural hypotension) OR (hypotension or low blood pressure) OR hypotension, orthostatic, OR hypotens*	32,720
S3	S1 OR S2	1,166,465
S2	(aging or ageing or elderly or older adults or seniors or geriatrics) OR (aged or older adults or geriatrics or senior) OR (seniors or elderly) OR (senescen* or senior*)	1,146,004
S1	(frailty or frail elderly or vulnerable elderly or vulnerability or functionally impaired elderly) OR frail elderly OR (frail* or pre frailty or pre-frail) OR (frailty or frail elderly)	42,569
**SCOPUS Search Strategy**		
**Search string**		**Results**
(( frailty OR frail OR frail* OR pre AND frail OR pre AND frailty OR frail AND elderly ) ) OR ( ( ageing OR aging OR aged OR agin* OR older OR elderly OR elder* OR older* ) ) AND ( ( orthostatic AND hypotension OR hypotension OR hypoten* OR blood AND pressure OR orthostat* ) ) AND ( ( postural AND transition OR postural AND challenge OR sit AND to AND sand OR lie-to-stand OR posture* OR position* ) )		**936**
PUBMED Search Strategy		
((((((((“frailty”[MeSH Terms] OR “frailty”[All Fields] OR “frailties”[All Fields] OR (“frail”[All Fields] OR “frails”[All Fields] OR “frailty”[MeSH Terms] OR “frailty”[All Fields] OR “frailness”[All Fields]) OR “frail*”[All Fields] OR “pre”[All Fields])) AND (“frail”[All Fields] OR “frails”[All Fields] OR “frailty”[MeSH Terms] OR “frailty”[All Fields] OR “frailness”[All Fields])) OR “pre”[All Fields])) AND (“frailty”[MeSH Terms] OR “frailty”[All Fields] OR “frailties”[All Fields])) OR (“frail”[All Fields] OR “frails”[All Fields] OR “frailty”[MeSH Terms] OR “frailty”[All Fields] OR “frailness”[All Fields])) AND (“aged”[MeSH Terms] OR “aged”[All Fields] OR “elderly”[All Fields] OR “elderlies”[All Fields] OR “elderly s”[All Fields] OR “elderlys”[All Fields])) OR (“aging”[MeSH Terms] OR “aging”[All Fields] OR “ageing”[All Fields] OR (“aging”[MeSH Terms] OR “aging”[All Fields] OR “ageing”[All Fields]) OR (“aged”[MeSH Terms] OR “aged”[All Fields]) OR “agin*”[All Fields] OR (“older”[All Fields] OR “olders”[All Fields]) OR (“aged”[MeSH Terms] OR “aged”[All Fields] OR “elderly”[All Fields] OR “elderlies”[All Fields] OR “elderlys”[All Fields] OR “elderlys”[All Fields]) OR “elder*”[All Fields] OR “older*”[All Fields])) AND (((((“orthostatically”[All Fields] OR “orthostatics”[All Fields] OR “orthostatism”[All Fields] OR “standing position”[MeSH Terms] OR (“standing”[All Fields] AND “position”[All Fields]) OR “standing position”[All Fields] OR “orthostatic”[All Fields]) OR “hypotension”[MeSH Terms] OR “hypotension”[All Fields] OR “hypotensions”[All Fields] OR “hypotensive”[All Fields])) OR “hypotensives”[All Fields] OR “hypotension”[MeSH Terms] OR “hypotension”[All Fields] OR “hypotensions”[All Fields] OR “hypotensive”[All Fields]) OR “hypoten*”[All Fields] OR (“blood”[MeSH Subheading] OR “blood”[All Fields] OR “blood”[MeSH Terms] OR “orthostat*”[All Fields]) AND (((((“postural”[All Fields] OR “posturally”[All Fields] OR “posture”[MeSH Terms] OR “posture”[All Fields] OR “postures”[All Fields] OR “postured”[All Fields] OR “posturing”[All Fields]) AND (“transit”[All Fields] OR “transited”[All Fields] OR “transiting”[All Fields] OR “transition”[All Fields] OR “transitional”[All Fields] OR “transitionals”[All Fields] OR “transitioned”[All Fields] OR “transitioning”[All Fields] OR “transitions”[All Fields] OR “transits”[All Fields])) OR (“postural”[All Fields] OR “posturally”[All Fields] OR “posture”[MeSH Terms] OR “posture”[All Fields] OR “postures”[All Fields] OR “postured”[All Fields] OR “posturing”[All Fields])) AND (“challenge”[All Fields] OR “challenged”[All Fields] OR “challenges”[All Fields] OR “challenging”[All Fields])) OR “sit-to-stand”[All Fields] OR “lie-to-stand”[All Fields] OR “postur*”[All Fields] OR “position*”[All Fields]))		**2209**

* The numbers in bold represent the final number that was included in the search for articles in each database.

## Appendix B. Preferred Reporting Items for Systematic Review and Meta-Analysis Protocols (PRISMA-P) 2015 Checklist

PRISMA-P (Preferred Reporting Items for Systematic review and Meta-Analysis Protocols) 2015 checklist: recommended items to address in a systematic review protocol *.

**Table:**

Section and Topic	Item No	Checklist Item	Location Where Item Is Reported
Administrative Information			
Title:			
Identification	1a	Identify the report as a protocol of a systematic review	1
Update	1b	If the protocol is for an update of a previous systematic review, identify as such	N/A
Registration	2	If registered, provide the name of the registry (such as PROSPERO) and registration number	PROSPERO - CRD42022268613
Authors:			
Contact	3a	Provide name, institutional affiliation, e-mail address of all protocol authors; provide physical mailing address of corresponding author	1
Contributions	3b	Describe contributions of protocol authors and identify the guarantor of the review	11
Amendments	4	If the protocol represents an amendment of a previously completed or published protocol, identify as such and list changes; otherwise, state plan for documenting important protocol amendments	N/A
Support:			
Sources	5a	Indicate sources of financial or other support for the review	11
Sponsor	5b	Provide name for the review funder and/or sponsor	11
Role of sponsor or funder	5c	Describe roles of funder(s), sponsor(s), and/or institution(s), if any, in developing the protocol	11
**Introduction**			
Rationale	6	Describe the rationale for the review in the context of what is already known	1, 2
Objectives	7	Provide an explicit statement of the question(s) the review will address with reference to participants, interventions, comparators, and outcomes (PICO)	5
**Methods**			
Eligibility criteria	8	Specify the study characteristics (such as PICO, study design, setting, time frame) and report characteristics (such as years considered, language, publication status) to be used as criteria for eligibility for the review	5, 6
Information sources	9	Describe all intended information sources (such as electronic databases, contact with study authors, trial registers or other grey literature sources) with planned dates of coverage	5
Search strategy	10	Present draft of search strategy to be used for at least one electronic database, including planned limits, such that it could be repeated	6, 7
Study records:			
Data management	11a	Describe the mechanism(s) that will be used to manage records and data throughout the review	8, 9
Selection process	11b	State the process that will be used for selecting studies (such as two independent reviewers) through each phase of the review (that is, screening, eligibility and inclusion in meta-analysis)	8
Data collection process	11c	Describe planned method of extracting data from reports (such as piloting forms, done independently, in duplicate), any processes for obtaining and confirming data from investigators	6, 7, 8
Data items	12	List and define all variables for which data will be sought (such as PICO items, funding sources), any pre-planned data assumptions and simplifications	7
Outcomes and prioritization	13	List and define all outcomes for which data will be sought, including prioritization of main and additional outcomes, with rationale	7, 8, 9
Risk of bias in individual studies	14	Describe anticipated methods for assessing risk of bias of individual studies, including whether this will be done at the outcome or study level, or both; state how this information will be used in data synthesis	8
Data synthesis	15a	Describe criteria under which study data will be quantitatively synthesised	8, 9
	15b	If data are appropriate for quantitative synthesis, describe planned summary measures, methods of handling data and methods of combining data from studies, including any planned exploration of consistency (such as I^2^, Kendall’s τ)	8, 9
	15c	Describe any proposed additional analyses (such as sensitivity or subgroup analyses, meta-regression)	9
	15d	If quantitative synthesis is not appropriate, describe the type of summary planned	N/A
Meta-bias(es)	16	Specify any planned assessment of meta-bias(es) (such as publication bias across studies, selective reporting within studies)	9
Confidence in cumulative evidence	17	Describe how the strength of the body of evidence will be assessed (such as GRADE)	9

* It is strongly recommended that this checklist be read in conjunction with the PRISMA-P Explanation and Elaboration (cite when available) for important clarification on the items. Amendments to a review protocol should be tracked and dated. The copyright for PRISMA-P (including checklist) is held by the PRISMA-P Group and is distributed under a Creative Commons Attribution Licence 4.0 [33].
